# Improving the interpretation of quality of life evidence in meta-analyses: the application of minimal important difference units

**DOI:** 10.1186/1477-7525-8-116

**Published:** 2010-10-11

**Authors:** Bradley C Johnston, Kristian Thorlund, Holger J Schünemann, Feng Xie, Mohammad Hassan Murad, Victor M Montori, Gordon H Guyatt

**Affiliations:** 1 Department of Clinical Epidemiology & Biostatistics, McMaster University, Hamilton, Ontario, Canada; 2 Department of Medicine, McMaster University, Hamilton, Ontario, Canada; 3 Programs for Assessment of Technology in Health Research Institute, Hamilton, Ontario, Canada; 4 Knowledge and Encounter Research Unit, Mayo Clinic, Rochester, Minnesota, USA

## Abstract

Systematic reviews of randomized trials that include measurements of health-related quality of life potentially provide critical information for patient and clinicians facing challenging health care decisions. When, as is most often the case, individual randomized trials use different measurement instruments for the same construct (such as physical or emotional function), authors typically report differences between intervention and control in standard deviation units (so-called "standardized mean difference" or "effect size"). This approach has statistical limitations (it is influenced by the heterogeneity of the population) and is non-intuitive for decision makers. We suggest an alternative approach: reporting results in minimal important difference units (the smallest difference patients experience as important). This approach provides a potential solution to both the statistical and interpretational problems of existing methods.

## Introduction

Health-related quality of life (HRQL) is increasingly recognized as an important outcome in randomized trials. Disease-specific HRQL instruments provide critical information because of their ability to detect small but important treatment effects [1,2]. Typically, for specific conditions, a number of disease-specific instruments are available. For example, there are at least five instruments available to measure HRQL in patients with chronic obstructive respiratory disease (COPD) (Chronic Respiratory Questionnaire, Clinical COPD Questionnaire, Pulmonary Functional Status & Dyspnea Questionnaire, Seattle Obstructive Lung Disease Questionnaire, St Georges Respiratory Questionnaire)[3].

Clinical trial investigators use different HRQL instruments for various reasons, including their familiarity with an instrument. This creates challenges for meta-analysts seeking summary estimates in systematic reviews of trials addressing the same or similar HRQL constructs. Choices include reporting summary estimates for each separate measurement instrument, or pooling across instruments. The former approach is less appealing in that it leaves the clinician with multiple imprecise estimates of effect.

A widely used approach to providing summary estimates across instruments - an approach endorsed by the Cochrane Collaboration - involves dividing mean differences between intervention and control in each study by the study's standard deviation (SD) and calculating what are called "standardized mean differences" (SMDs) or "effect sizes". Ultimately, systematic reviews using this approach will present the magnitude of treatment effects as SD units (e.g., pooled estimate 0.4 SD units)[4].

This approach provides a single pooled estimate of treatment effect but leaves two problems. One problem is that if the heterogeneity of patients is different in different studies, the SD will vary across studies. Therefore, given the same true difference in HRQL between intervention and control groups, trials with more heterogeneous patients and similar scores on the HRQL instrument of interest will show apparently - but spuriously - smaller effects than trials enrolling less heterogeneous patients.

The second problem is that interpretation of the magnitude of effect when represented as SD units is challenging. Although rules of thumb - the most frequently used guide tells us that an effect size of 0.2 represents a small difference, 0.5 a moderate difference, and 0.8 a large difference [5] - are available, they have limitations. They are to an extent arbitrary, and do not intuitively resonate with either clinicians or patients [6].

One strategy to address similar problems in interpretation of results from individual trials that report on HRQL measures involves the minimal important difference (MID) [7,8]. The MID is defined as "the smallest difference in score in the outcome of interest that informed patients or informed proxies perceive as important, either beneficial or harmful, and which would lead the patient or clinician to consider a change in the management" [9]. A variety of statistical and anchor-based approaches to ascertaining the MID of individual instruments are available [10].

If the MID has been established for two or more instruments, systematic review authors could report the results of each study in "MID units" instead of SD units (for both individual studies and pooled effects). Standardization using MIDs may provide a uniform metric that both circumvents the fragile assumption regarding similar variability in study populations across trials that is required for the SMD and facilitates interpretation by both clinicians and patients. In the remainder of this article, we will illustrate both the current and proposed methods using data from a systematic review of respiratory rehabilitation in COPD [11]. Although we focus on disease specific HRQL, the method can be applied to any meta-analysis of RCTs that employ patient important continuous outcome measures.

## Standard methods for meta-analysis of HRQL measures

Health Related Quality of Life scores are typically treated as continuous. In meta-analysis of continuous data, the mean difference (MD), or the "difference in means" is the measure of the absolute difference between the mean value in each arm in a parallel group clinical trial. When outcome measurements in all trials are made on the same scale, a well-established inverse variance meta-analysis method can be used to combine results across trials and obtain a pooled MD [4].

When investigators have relied on different instruments measuring the same or similar construct, it is necessary to transform or standardize the trial results to a uniform scale before they can be combined in a meta-analysis. The common approach to the problem is to calculate the SMDs for each trial (i.e., the trial MD divided by its SD) and pool across trials.

## MID method for meta-analysis of different HRQL instruments

A potential solution to the limitations of SMD is to substitute the MID for the usual denominator of the SMD, the SD. That is, we divide the MD by the MID that was established for the instrument used in the trial. As a result, rather than obtaining an estimate in SD units, we obtain an estimate in MID units.

When we standardize by dividing the MD by the MID, we alter the scale on which we are performing our meta-analysis. In doing so, we also need to account for the changes that the standardization has on the standard error and weights associated with each standardized trial outcome. In the accompanying appendix (additional file 1) we derive the formulas for the variance and standard error of the pooled MD, and provide formulas for the pooling of results.

## Application of the method

A Cochrane review of respiratory rehabilitation for COPD included 31 trials [11] of which 16 employed two widely used disease-specific HRQL instruments: the Chronic Respiratory Disease Questionnaire (CRQ),[12] and the St. Georges Respiratory Questionnaire (SGRQ) [13]. Extensive evidence supports the validity and responsiveness of both these instruments, and both have established MIDs [9,14]. The authors of the systematic review calculated separate pooled MD estimates for the trials using the instruments' individual "natural units" (i.e., the 7-point scale for the CRQ and the 100 point scale for the SGRQ [11]).

## Pooled estimates for CRQ and SGRQ

Using data from the systematic review, we calculated MDs (and 95% Confidence Intervals) separately for each of the four domains of the CRQ and each of the three domains of the SGRQ, as well as an overall score for each instrument. For the CRQ, most of the included trials did not report the overall mean (SD). To resolve this, we generated the overall mean (SD) for each trial using the domain data provided by the Cochrane review (see additional file 1).

**Figure 1:**
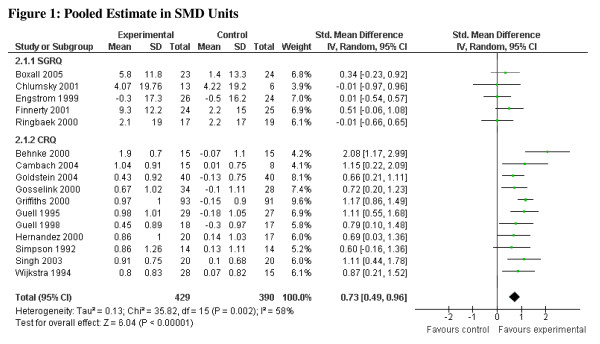
**Pooled Estimate in SMD Units**.

For the CRQ, the pooled MD for each of the domains (dyspnea, emotional function, fatigue, and mastery) as well as the total score exceeded the MID, as did the lower limit of the confidence interval for each domain (0.5 points difference on the 7-point scale) [9]. For the SGRQ, the pooled MD for each of the domains as well as the total score exceeded the MID (4 points difference on the 100-point scale) [14]. The confidence interval for each of the domains and the overall pooled MD, however, included values less than the SGRQ's MID of 4.0 (see Table 1). The CRQ and SGRQ estimates, pooled separately, include one study [15] that employed both instruments. The pooled estimates in SD units are, for the CRQ, 0.96 (95% CI 0.76, 1.16), and for the SGRQ, 0.36 (95% CI 0.12, 0.60). Combining all studies yields an overall pooled estimate in SD units of 0.73 (95% CI 0.49, 0.96), I^2 ^= 58% (Figure 1). To avoid double counting, for the overall pooled estimate in SD units (and below for MID units), we included only the CRQ results for Griffiths et al [15]. Although both the SGRQ and CRQ have been widely used, and have demonstrated validity and responsiveness in various settings; the reason we chose the CRQ as the reference instrument was the stronger evidence supporting the MID and evidence of superior responsiveness [9,16].

**Table 1:** Pooled Mean Differences from Trials Included in Cochrane Review

CRQ	Point estimate (95% Confidence Interval)
Dyspnea	1.06 (0.85, 1.26)

Emotional Function	0.76 (0.52, 1.00)

Fatigue	0.92 (0.71, 1.13)

Mastery	0.97 (0.74, 1.20)

Overall	0.94 (0.57, 1.32)

**SGRQ**	

Activities	-4.78 (-1.72, -7.83)

Impacts	-6.27 (-2.47, -10.08)

Symptoms	-4.68 (0.25, -9.61)

Overall	-6.11 (-3.24, -8.98)

Note: negative scores on the SGRQ indicate improvement

## Results in MID units

**Figure 2:**
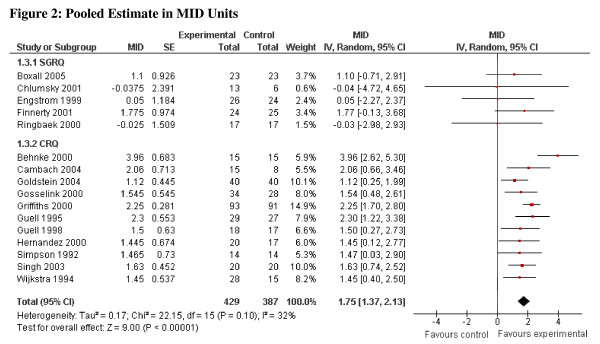
**Pooled Estimate in MID Units**.

Applying the new method the pooled estimates in MID units are, for the CRQ, 1.86 (95% CI, 1.45 to 2.27) and for the SGRQ, 1.53 (95% CI, 0.81 to 2.24). For both measures the common effect size exceeded 1.0 indicating that the intervention effect is, on average, appreciably greater than the MID. With respect to the lower confidence interval around the common effect size, the CRQ results exceeded 1.0, whereas the SGRQ did not. We can thus be confident, on the basis of the studies using the CRQ, that the mean effect exceeds the MID whereas, for the SGRQ we cannot. Combining all studies in MID units yields an overall pooled estimate of 1.75 (95% CI, 1.37 to 2.13), I^2 ^= 32% (Figure 2).

## Interpreting MID unit results

The point estimate in MID units suggests a large effect (approaching 2 MIDs) and the lower 95% CI is greater than 1, suggesting that it is implausible that the mean effect is less than the MID (Figure 2). However, reporting results in MID units risks naïve misinterpretation: above 1 MID treatment has important benefits for all patients, and below 1 for none. Even if the pooled estimate lies between 0 and 1 (or 0 and -1), treatment may have an important impact on many patients [17]. We suggest the following guide for interpretation: if the pooled estimate is greater than 1 MID, and one accepts that the estimate of effect is accurate, many patients may gain important benefits from treatment. If the estimate of effect lies between 0.5 and 1.0, the treatment may benefit an appreciable number of patients. As the pooled estimate falls below 0.5 MID it becomes progressively less likely that an appreciable number of patients will achieve important benefits from treatment.

## Strengths of the method

The major strength of our method is that it avoids the problems associated with heterogeneity of between-study variances (as a result of using the SD to calculate the SMD). The MID unit approach prevents introducing inconsistency depending on the SD of each included trial and provides results that are likely to facilitate intuitive interpretation by clinicians and patients. Of interest, the statistical heterogeneity as measured by the I^2 ^statistic decreased (from 58% to 32%) by reporting results in MID units as opposed to SD units. Future studies involving formal simulation techniques might consider evaluating I^2 ^estimations when considering SD units vs MID units.

## Limitations of the method

Our method requires that previous investigations have generated an estimate of the MID; this is true for only a limited number of HRQL measures. Nevertheless, MIDs are being increasingly established for instruments used to evaluate common illnesses [18,19]. If an anchor-based MID has not been established, distribution-based methods might provide a reasonable alternative for MID estimation [20]. Because one or more measures of variability are almost always available, distribution-based MIDs are relatively easy to generate [21]. Nevertheless, the circumstances in which distribution-based methods concur with anchor-based methods, and the ideal distribution-based method to use, remains unclear.

Even if the MID is available, application of the method in particular instances may present challenges. In the example we have used, the CRQ was not originally developed to provide an overall summary score, and for this reason the majority of included trials calculated estimates for each domain separately. We felt comfortable with this strategy because previous work has demonstrated that an overall score is sensible and likely remains valid and responsive [22]. An additional limitation is that, as described above, MID units are vulnerable to naïve, oversimplified interpretation. Vulnerability to misinterpretation is not, however, unique to the MID approach. We have suggested a rule-of-thumb guide to the interpretation of MID units, a guide that is somewhat arbitrary. Repeated experience using MID units, in particular examining the relation between MID unit effect and the difference in proportion of patients demonstrating an improvement of at least 1 MID unit in intervention and control groups, will further enhance and refine the interpretability of the MID approach.

## Conclusion

Systematic reviews and meta-analyses of randomized trials that employ HRQL instruments provide the least biased and most precise summary estimates of the impact of interventions on patients' lives. When, however, individual randomized trials use different measurement instruments for the same construct, existing methods for combining across studies are plagued by statistical and interpretational limitations. The MID approach provides a potential solution to the limitations of the existing methods.

## Competing interests

HJS and GHG are authors of the Chronic Respiratory Questionnaire.

## Contributions

BCJ participated in design of the study, data extraction, data analysis, interpretation of the results and drafted the manuscript. KT participated in the design of the study, developed the statistical framework for data analysis and participated in the interpretation of the results. HJS, FX, MHM, VMM participated in the design of study and interpretation of the results. GHG participated in the design of study, developed the statistical framework for data analysis and participated in the interpretation of the results. All authors critically revised the article and approved the version to be published.

## Supplementary Material

Additional file 1 **Total CRQ score formulas and MID units formulas.** Total CRQ score formulas: Standard errors and standard deviations for total CRQ scores. MID units formulas: Pooling MID standardized mean differences Click here for file
