# Supplementary material for: Improving the interpretation of quality of life evidence in meta-analyses: the application of minimal important difference units
Source: Health Qual Life Outcomes. 2010 Oct 11;8:116. doi: 10.1186/1477-7525-8-116 (PMC2959099; doi:10.1186/1477-7525-8-116)
Supplement: Additional file 1 — Total CRQ score formulas and MID units formulas. Total CRQ score formulas: Standard errors and standard deviations for total CRQ scores. MID units formulas: Pooling MID standardized mean differences [file 1477-7525-8-116-S1.DOC]

**Total CRQ score formulas: Standard errors and standard deviations for total CRQ scores**

To calculate the standard deviations and standard errors for the total CRQ treatment group mean, and the total CRQ control group mean, and the total CRQ mean difference, we assumed a correlation of *ρ*=0.5. Let *m_EF_,* *m_EE_*, *m_EM_* and *m_ED_* denote the means for the Fatigue(F), Emotional(E), Mastery(M) and Dyspnea(D) domains in the experimental group, let *m_CF_,* *m_CE_*, *m_CM_* and *m_CD_* denote the means for the four domains in the control group. Let *x* denote the treatment group (either E or C). We calculate the total variance for the average of treatment means across domains using the formula

$$\begin{matrix} Var\left(\frac{m_{XF}+m_{XE}+m_{XM}+m_{XD}}{4}\right){}= \\ \frac{1}{16}\left(\sum_{i=\{F,E,M,D\}} Var(m_{xi})+\sum_{i=\{F,E,M,D\}} \sum_{i\neq j} Cov\left(m_{xi},m_{xj}\right)\right) \end{matrix}$$

Where we use that

$$\begin{matrix} Cov\left(x_{i},x_{j}\right)=\rho\sqrt{Var\left(x_{i}\right)Var\left(x_{j}\right)} \\ =0.5\sqrt{Var\left(x_{i}\right)Var\left(x_{j}\right)} \end{matrix}$$

We obtain the standard error by taking the squareroot of the variance

$$\begin{matrix} SE_{x}=SE\left(\frac{m_{XF}+m_{XE}+m_{XM}+m_{XD}}{4}\right){}= \\ \sqrt{Var\left(\frac{m_{XF}+m_{XE}+m_{XM}+m_{XD}}{4}\right)} \end{matrix}$$

and the standard deviation by multiplying the standard error by the squareroot of the number of patients in the treatment group, n_x_

$$\begin{matrix} SD_{x}=SD\left(\frac{m_{XF}+m_{XE}+m_{XM}+m_{XD}}{4}\right){}= \\ \sqrt{n_{x}}\cdot SD\left(\frac{m_{XF}+m_{XE}+m_{XM}+m_{XD}}{4}\right) \end{matrix}$$

Now let *m_F_,* *m_E_*, *m_M_* and *m_D_* denote the mean differences for the four domains. We obtain the standard error of the average of the mean differences across domains using the formula

$$SE\left(\frac{m_{F}+m_{E}+m_{M}+m_{D}}{4}\right){}=\sqrt{\frac{SD_{E}^{2}}{n_{E}}+\frac{SD_{C}^{2}}{n_{C}}}$$

MID units formulas: Pooling MID standardized mean differences

Assume that a trial reports a MD on some disease-specific HRQL instrument X, and assume that the minimally important different for instrument X, **MID_X_, has been established. T**he estimated MD is a random variable. **If we standardize this random variable by dividing it by the MID_X_, we get a new random variable, MD/MID_X_.** We know from basic probability theory that because MID_X_ is a constant, the variance of **MD/MID_X_** is given by

$$Var\left(\frac{MD}{MID_{X}^{2}}\right)=\frac{Var\left(MD\right)}{MID_{X}^{2}}$$

That is, the variance of the mean difference divided by the square of the MID. Further, the standard error of **MD/MID_X_ is given by**

$$SE\left(\frac{MD}{MID_{X}^{2}}\right)=\sqrt{\frac{Var\left(MD\right)}{MID_{X}^{2}}}=\frac{SE\left(MD\right)}{MID_{X}}$$

Now suppose a meta-analysis included *k* trials. The first *j* trials use disease-specific instrument A, and the last *k-j* trials use disease-specific instrument B. Let MD_i_ denote the mean difference observed in trial *i*, let MID_A_ denote the minimally important difference established for instrument A, and let MID_B_ denote the minimally important difference established for instrument B. Further, let *m_i_* denote the MID standardized effect for trial *i.*

To pool results across trials using MIDs we must first estimate the *m_i_* and its associated variance for all trials. For *i=1, …, j* we have

$$m_{i}=\frac{MD_{i}}{MID_{A}}andVar\left(m_{i}\right)=\frac{Var\left(MD_{i}\right)}{MID_{A}^{2}}$$

and for *i=j+1, …, k* we have

$$m_{i}=\frac{MD_{i}}{MID_{B}}andVar\left(m_{i}\right)=\frac{Var\left(MD_{i}\right)}{MID_{B}^{2}}$$

By defining the trial weights as *w_i_=Var(m_i_)^-1^,* we can use the fixed-effect model inverse variance method to pool the MID-standardized mean differences using the formula

$$\hat{m}=\left(\sum_{i=1}^{k} w_{i}\cdot m_{i}\right)/\left(\sum_{i=1}^{k} w_{i}\right)$$

Where $\hat{m}$denotes the pooled MID-standardized mean difference. The standard error of $\hat{m}$can be calculated using the formula

$$se(\hat{m})=1/\left(\sum_{i=1}^{k} w_{i}\right)$$

and confidence intervals can subsequently be derived. Pooling of MID-standardized mean differences is naturally extended to the random-effects model using weights *w_i_=(Var(m_i_)+τ^2^)^-1^,* where *τ^2^* denotes the between-trial variance.
